# Low-dose ruxolitinib plus steroid in severe SARS-CoV-2 pneumonia

**DOI:** 10.1038/s41375-020-01087-z

**Published:** 2020-11-10

**Authors:** A. D’Alessio, P. Del Poggio, F. Bracchi, G. Cesana, N. Sertori, D. Di Mauro, A. Fargnoli, M. Motta, C. Giussani, P. Moro, G. Vitale, M. Giacomini, G. Borra

**Affiliations:** 1 COVID Medical Department, Policlinico S.Marco Gruppo San Donato University and Research Hospital, Zingonia Bergamo, Italy; 2 Intensive Care Unit, Policlinico S.Marco Gruppo San Donato University and Research Hospital, Zingonia Bergamo, Italy; 3 Policlinico S.Marco Gruppo San Donato University and Research Hospital, Zingonia Bergamo, Italy

**Keywords:** Diseases, Infectious diseases, Therapeutics

## To the Editor:

SARS-CoV-2 is a biphasic illness characterized by a first flu-like phase, followed by a pulmonary and systemic disease, in which a dysregulated cytokine storm may lead to acute respiratory distress (ARDS) and death [1]. JAK-STAT inhibitors block the common pathway of cytokine activation, may reduce the over-exuberant inflammatory reaction and decrease mortality [2, 3].

Ruxolitinib is a JAK 1 and 2 (Janus Kinase) inhibitor used in the treatment of myelofibrosis, policytemia vera and hemophagocytic lymphohistiocytosis, which is characterized by a cytokine derangement similar to what observed in SARS-CO-V2 infection [4].

We performed a non-randomized clinical study on the effect of ruxolitinib in patients with severe COVID-19 pneumonia not requiring mechanical ventilation at baseline (group A), comparing them to a control group of patients with the same clinical and radiological characteristics and hospitalized in the same time period (group B). All patients over age 18, admitted to our Hospital from March 13, 2020 to April 13, 2020 and eligible to ruxolitinib Managed Access Plan (MAP) were evaluated. The MAP program for ruxolitinib was registered by Novartis (https://clinicaltrials.gov/ct2/show/NCT04337359), approved by the Italian Ministry of Health and by the ethical review board of our institution. Inclusion criteria were a nasopharyngeal swab positive for SARS-CoV2 by polymerase chain reaction, pulmonary infiltrates typical for interstitial pneumonia on Chest CT and respiratory frequency >30/min or oxygen saturation equal or lower than 93%. We excluded patients with chronic comorbidities or neoplastic disease with <1 year life expectancy, documented bacterial superinfections, advanced dementia, previous treatment with anti-interleukin 1 or anti-interleukin 6 inhibitors. Ruxolitinib was administered orally at a dose of 5 mg twice daily for 7 days and then tapered to 5 mg daily to complete a 10-day course of treatment. In addition to ruxolitinib all patients received methyl-prednisolone 1 mg/kg intravenously for 3 days followed by 0.5 mg/kg for 5 days and then oral prednisone, which was slowly tapered in the course of 2 weeks. Concomitant antiviral therapy such as hydroxychloroquine, lopinavir/ritonavir, remdesivir was not permitted during treatment with ruxolitinib. The control group was chosen among the patients admitted to our hospital in the same period for COVID-19 pneumonia and hospitalized in a ward with the same intensity of care and who did not apply for Ruxolitinib MAP. Both treated and control groups were comparable, with the exception of comorbidities, which were slightly over-represented in the control group, although not significantly (Table 1). Primary outcomes were defined as clinical recovery without mechanical ventilation, admission to ICU for mechanical ventilation and death. Patients were considered clinically recovered if they had been afebrile for at least 3 days before hospital discharge and with an oxygen saturation of at least 95%. Both overall survival and survival free of unwanted outcomes—i.e., death and ICU admission—were evaluated as intention to treat by means of Kaplan–Meyer analysis. As a secondary outcome we considered a reduction of the inflammatory response, defined as absence of fever and at least a 30% decrease of CRP levels at the second clinical observation—i.e., 3–10 days after hospital admission. Patients were censored at the time of live discharge from the hospital. Statistical analysis was performed with MedCalc software (18.11.6 version).

**Table 1:** Baseline characteristics of the patients.

	Group A (32)	Group B (43)	*p*
Mean age in years (95% C.I.)	67.5 (63–71)	67.8 (64–71)	*p* = 0–65^
Male Sex *n* (%)	22 (69)	30 (69)	*p* = 1.00^*^
Mean BMI (±SD)	25.4 (3.74)	25.03 (3.42)	*p* = 0.37^
Oxygen support (on admission)			
None *n*. (%)	11 (34)	16 (37)	*p* = 0.80^*^
Low flow oxygen (<10 l/min)	6 (19)	5 (11)	
High flow oxygen (>10 l/min)	15 (47)	22 (51)	
Coexisting conditions			
Any condition n (%)	15 (47)	25 (58)	*p* = 0.33^*^
Hypertension	15 (47)	20 (46)	
Diabetes	4 (13)	8 (18)	
Others		4 (9)	
Chest CT n (%)			
<50% lung involvement	17 (53)	20 (46)	*p* = 0.57^*^
>50% lung involvement	15 (47)	23 (53)	
Median laboratory values:			
Oxygen saturation % (median and 95% C.I)	91.7 (90–92)	91.9 (91–92)	*p* = 0.29^^
Creatinine mg/dL (median and 95% C.I.)	1.2 (1.09–1.30)	1.2 (1.0–1.43)	*p* = 0.75^^
PCR mg/dL^§^ (median and 95% C.I.)	14.2 (7.8–17.9)	12.2 (10–15.1)	*p* = 0.90^^
D–Dimer^§§^ ng/ml (median and 95% C.I.)	1.3 (0.83–1.92)	1.07 (0.70–1.70)	*p* = 0.65^^
Total Lymphocyte count (x 10^3^)	0.9 (0.65–1–0)	0.9 (0.7–1.1)	*p* = 0.77^^
ISTH–SIC score^**^ No (%)			
0	21 (66)	26 (60)	(0 + 1 vs 2) *p* = 1.00*
1	10 (31)	14 (32)	
2	1 (3)	2 (5%)	
3–4	–	–	

^*^Chi Square ^ Student *t* test ^^ Mann–Whitney rank-sum test ^§^normal values <0.5 mg/dL ^§§^normal values <0.4 ng/ml.

^**^International Society of Thrombosis and Haemostasis Sepsis Induced Coagulation Score [Iba et al. J Thromb Haemost. 2019; 17(11):1989–1994].

Among the 93 patients admitted from March 13 to April 13, 32 (35%) were enrolled into the MAP program for ruxolitinib, 43 (46%) served as a control group and 18 (19%) were excluded from the analysis because they did not meet the inclusion criteria of this study. During the hospitalization 23 patients of group B (67%) received hydroxychloroquine, 13 (30%) both hydroxycloroquine and lopinavir/ritonavir and, 11 (25%) methyl-prednisolone at the dose of 0.5–1 mg/kg for 3–10 days. Two patients of group A (6.3%) and three of group B (6.9%) received enoxaparin at the therapeutic dose of 100 IU/Kg twice a day, all the other patients were treated prophylactically with 100 IU/kg daily.

**Fig. 1:**
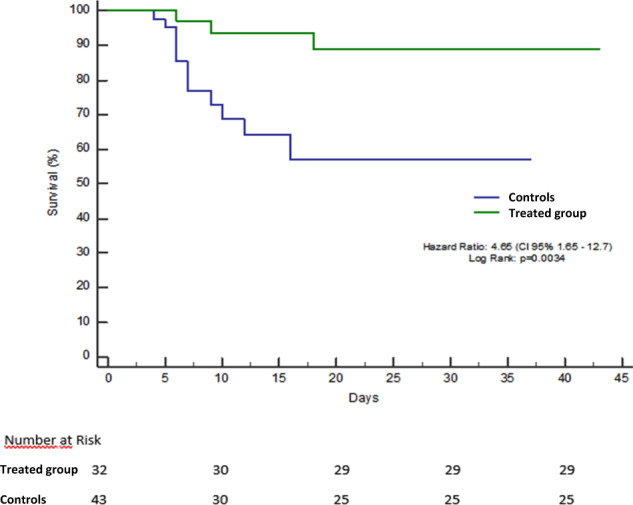
Overall survival in patients with severe COVID-19 pneumonia treated with ruxolitinib plus steroid (group A) compared to controls (Group B): Kaplan Meyer curves. The day of hospitalization was considered as time 0 in the *y* axis. Patients were censored at the time of live discharge from the hospital. No patient was lost at follow-up.

At the end of the study, all patients had reached the established endpoints. Twenty-four patients of group A (75%) were considered clinically recovered without admission to the ICU, five (16%) were transferred to the ICU, mechanically ventilated and clinically cured and three patients (9%) died, one of whom in the ICU. Ruxolitinib was withdrawn in all five patients admitted to the ICU, while steroid was continued as initially planned. No rebound of inflammation was observed in these patients. On the contrary only 27 patients of group B (63%) were considered clinically recovered, three (7%) were transferred to the ICU and clinically cured and 13 died (30%), three of whom in the ICU. Kaplan–Meyer estimates of the percentage of patients who were alive and clinically recovered at the end of follow-up were 89.1% (SE ± 10.3) for group A and 57.1% for group B (SE ± 6.1), as represented in Fig. 1 (*p* = 0.0034). Likewise the percentage of patients free of unwanted outcome at the same time point was 45% (SE 16.4) for group A and 22.9% (SE 17.2) for group B (*p* = 0.0052). The percentage of patients who reached the secondary endpoint was also significantly higher in the treated than in the control group: 28 patients of group A (87%) vs 10 of group B (23%), *p* = 0.0001. At the second clinical observation median CRP levels decreased from 14.6 (range 1.8–30) to 1.33 (range 0.05–22) mg/dL in group A, while remained stable in group B: 11.53 mg (range 1.8–29) and 12 (range 0.1–32) mg/dL. The difference between the two groups was highly significant (Mann–Whitney Rank Sum test *p* = 0.0001). We did not observe any major adverse event related to the use of steroid nor to ruxolitinib.

The patients included in our study had severe, hypoxemic COVID-19 related pneumonia, were admitted to medical wards and yet did not require mechanical ventilation. These patients represent a “gray zone” in which a downregulation of the over-exuberant immunity may prevent the progression of lung damage and avoid intubation. The time window to start anti-inflammatory drugs is very important because an early administration could enhance viral replication, while a late treatment could exacerbate the immunological exhaustion caused by the prolonged cytokine storm. We used an association of steroid and a JAK-1 and 2 inhibitor (ruxolitinib) as immunomodulatory drugs. The rationale was to enhance the broad immunosuppressant effect of corticosteroids with a specific multi-cytokine inhibitor such as ruxolitinib. JAK-STAT signaling is involved in the intracellular pathways of many inflammatory proteins and its inhibition may lead to upstream down-regulation of IL-6, TNF-α, IL-1β, IL-2, IL-8 [5]. This extended action could entail a better control of the hyper-inflammatory syndrome than that achieved by single cytokine inhibitors like tocilizumab or anakinra. The use of a dual JAK1 and 2 inhibitor like ruxolitinib may also impact on T cell proliferation and activity, which play an important role in the cytokine storm, an effect mediated by the JAK-1 receptor and not achievable by selective JAK-2 inhibitors [6]. Our main concern with the use of JAK-STAT inhibitors was the risk of enhancing viral replication and the occurrence of opportunistic infections, and for that reason we used half of the approved dose of ruxolitinib for hematologic diseases. JAK-STAT inhibitors may enhance viral replication by inhibiting interferon alpha production, but have also direct antiviral activity [7], although this effect can be achieved by ruxolitinib only at extremely high doses [8]. Baricitinib, another JAK1-2 inhibitor with stronger affinity for the AAK1 receptor down regulating Chlatryn mediated viral entry [8], has a greater antiviral activity and theoretically could be the drug of choice. However, it should be used with great caution in COVID-19 patients because of its propensity for arterial thrombosis [9]. COVID-19 related pneumonia is characterized by a severe pulmonary intravascular coagulopathy [10, 11] and the use of baricitinib could trigger or aggravate the immune mediated thrombosis.

We found a significant reduction in mortality and no significant adverse event in treated patients compared to controls. These favorable results were achieved through an attenuation of the systemic inflammatory response, since we detected a faster decline in CRP levels and disappearance of fever in treated patients. It is possible that steroids may have played a synergistic role with ruxolitinib in dampening the immune over-reactivity. In a recent large randomized trial steroids significantly reduced mortality in patients with COVID-19 pneumonia necessitating oxygen supplementation or mechanical intubation [12], but the reduction in mortality observed in our study with combination therapy was much greater. It is also worth noting that, in spite of a significant proportion of controls receiving steroids (25%), the strength of the survival benefit was not weakened, suggesting that ruxolitinib was the most effective agent of the association. It should be explored in further randomized trials whether steroids provide real additive benefit to ruxolitinib or not. We are at the dawn of a new era in the treatment of COVID-19 and the proper timing, selection and judicious use of immunosuppressive drugs together with antiviral therapy [13, 14] could hopefully reduce the high death toll observed in this dreadful disease.
